# A 25-year cohort study of the prevalence of deja vu, jamais vu and synesthesia phenomena in Rudn university students

**DOI:** 10.1192/j.eurpsy.2026.10924

**Published:** 2026-07-31

**Authors:** M. S. Artemieva, R. Suleimanov, I. Danilin, A. Lazukova

**Affiliations:** Peoples’ Friendship University of Russia named after Patrice Lumumba, Moscow, Russian Federation

## Abstract

**Introduction:**

Scientific literature from the 19th and 20th centuries indicates that approximately two-thirds of all people reported experiencing déjà vu and various similar states.

**Objectives:**

The aim of this 25-year study was to investigate the prevalence and incidence of deja vu, jamais vu and synesthesia symptoms in modern society.

**Methods:**

Fifth-year medical students of RUDN University aged 22-24 years +/- 2.4 participated in this study annually from 2001 to 2025. A total of 2860 students were included in the study.

**Results:**

The prevalence of déjà vu at the beginning of the observation (2001–2004) was 82% (95% CI: 79–85%), then consistently increased by 2015 reaching 98.5% (95% CI: 97–99%) and remains at this level to this day. For most respondents, déjà vu occurs quite rarely (from 2 to 10 times over many years), mainly in childhood and adolescence.

A consistent increase in the incidence of synesthesia in students was noted, as a stable feature that is not subject to significant changes and persists throughout life, from 12.6% (95% CI: 11-16%) in 2000-2015 to 22.3% (95% CI: 20-25%) in 2016-2025 (χ2 = 41.7, p < 0.001).

The jamais vu phenomenon was relatively rare: in 2001-2017, the frequency was 3% (95% CI: 2-5%), and in 2018-2025. increased to 5% (95% CI: 3–8%) (χ^2^=6.7, p<0.01). Lifetime prevalence of jamais vu remained consistently low – 1–3 cases in 96% of respondents who answered positively.

**Conclusions:**

This study demonstrates a significant increase in the incidence of deja vu, jamais vu and synesthesia over the past 25 years and requires further investigation.

**Disclosure of Interest:**

None Declared

